# The causal association between insomnia and cognitive decline: A 2-sample, 2-step multivariable Mendelian randomization study

**DOI:** 10.1097/MD.0000000000043417

**Published:** 2025-07-18

**Authors:** Zilin Wang, Zihan Qu, Jindan Zhang, Yuqing Song, Jiawei Yin, Hongshi Zhang

**Affiliations:** a School of Nursing, Changchun University of Chinese Medicine, Changchun, China.

**Keywords:** cognitive impairment, insomnia, Mendelian randomization

## Abstract

While there have been many observational studies to date exploring the potential causal nature of any relationship between insomnia and cognitive decline, the available evidence remains contradictory. As such, in this study, a 2-sample Mendelian randomization (MR) study was performed using publicly accessible genome-wide association study data in order to clarify the mediating effects of several variables on this link. Results were validated by performing sensitivity analyses including the Cochran Q test, the MR-Egger intercept test, the Mendelian Randomization Pleiotropy RESidual Sum and Outlier, MR Radial, and leave-one-out analyses. Based on this 2-sample MR approach, genetically predicted insomnia was found to be negatively causally associated with cognitive function (β, −0.015 [95% CI, −0.026 to −0.004]; *P* = .006), with this causal link remaining intact following Bonferroni correction. Risk factors relevant to cognitive function including body mass index, respiratory tract infections (RTIs), blood metabolites, and immune cells were analyzed as potential mediating factors, ultimately leading to the identification of RTIs as a significant mediator of the causal link between insomnia and cognitive function (*P* < .05), mediating 15.4% of this effect. Insomnia is, thus, closely associated with impaired cognition, with RTI playing a role in the interplay between the 2. Developing effective approaches to the early treatment of insomnia, together with a focus on RTIs, may, thus, improve cognition both directly and indirectly through reductions in RTI incidence.

## 1. Introduction

Sleep is essential for maintaining cognitive function, as it supports processes such as toxin clearance, neural plasticity, and memory consolidation. However, chronic insomnia disrupts these mechanisms and is increasingly recognized as a risk factor for cognitive decline, particularly among aging populations.^[1]^ Almost 50% of adults over 60 years of age, however, report difficulties maintaining or initiating normal sleep patterns, with upward of 30% experiencing chronic insomnia and even higher rates having been reported among older populations.^[2]^ Insomnia is a condition characterized by the inability to fall asleep or remain asleep, together with daytime sleepiness, impaired daytime functioning, and associated distress.^[3]^ With the progressive aging of the global population, roughly 25.1% of the total world population will be over the age of 60 years by 2050, and this proportion is expected to reach 34.6% by 2100.^[4]^ Dementia and cognitive impairment are persistent global public health challenges. While the preservation of appropriate cognitive function is essential for the maintenance of independence when conducting activities of daily living, mild cognitive impairment, which affects >22% of adults aged ≥65 years, is a precursor to dementia, with ≈15% of patients progressing to dementia within 2 years.^[5]^ Owing to a lack of efficacious interventional strategies, the only viable approaches to delaying cognitive decline at present focus on mitigating associated risk factors and developing new protective strategies.

A growing number of studies suggest that insomnia can contribute to declining cognitive function. Relative to individuals not affected by insomnia, patients with long-term insomnia tend to exhibit more rapid cognitive decline together with issues including impaired memory, attention, and executive function.^[6]^ Sleep plays an important role in the clearance of toxins including β-amyloid proteins from the brain, and poor sleep quality can adversely affect brain blood flow and disrupt patterns of brain activity conducive to attention and memory, contributing to cognitive decline.^[7]^ In an 11-year longitudinal study, however, insomnia was not found to be associated with a risk of dementia, with researchers instead discovering that insomnia was unexpectedly linked to better cognitive function and a lower risk of dementia.^[8]^ However, most existing studies use small samples, cross-sectional designs, and insufficient adjustment for confounders; consequently, residual confounding remains and causal relationships cannot be established. Further research is required to clarify these associations. Potential pathways that may mediate the relationship between insomnia and cognitive function also remain poorly understood at present.

In order to more fully explore potential mediators of this link, several risk factors known to impact cognitive function, including body mass index (BMI), immune cells, respiratory tract infections (RTIs), and blood metabolites, were herein selected to screen for potential mediating factors in order to provide a foundation for future research. The selection of potential mediators, BMI, RTI, blood metabolites, and immune cells, was based on their established roles in both insomnia and cognitive decline. The study emphasizes that BMI may serve as a risk factor for insomnia and neurodegeneration through mechanisms involving metabolic dysregulation and chronic inflammation.^[9]^ RTIs, particularly pneumonia, are associated with systemic inflammation and the production of neurotoxic amyloid-beta and tau proteins, thereby accelerating cognitive deterioration.^[10]^ Blood metabolites (e.g., glucose and lipids) and immune cells (e.g., cytokines and T cells) are implicated in neuroinflammation and the disruption of the blood-brain barrier, processes that are further exacerbated by insomnia.^[11]^

Building upon existing literature, this study hypothesizes that insomnia contributes to cognitive decline through a mediating pathway involving RTI, particularly pneumonia. The proposed model suggests that insomnia-induced inflammation may increase susceptibility to RTI, which, in turn, exacerbates cognitive deterioration.

Mendelian randomization (MR) analyses allow for the use of single-nucleotide polymorphisms (SNPs) as instrumental variables (IVs) when assessing causal associations between a given exposure and a particular outcome, thereby helping to avoid the potential for confounding effects of reverse causality that can adversely affect conventional observational studies.^[12]^ While past MR studies have demonstrated that insomnia is causally related to the incidence of cognitive impairment, there have been insufficient efforts to identify potential mediators of this relationship or how different risk factors may interact in this context. As such, this study made use of genome-wide association study (GWAS) data to explore the effects that insomnia has on cognitive decline with a 2-sample MR approach, quantifying mediating effects for multiple risk factors with a multivariate MR (MVMR) approach in order to offer stronger evidence in support of the causal association between insomnia and cognitive decline. MVMR, which simultaneously accounts for multiple exposures, offers a novel framework to disentangle direct and indirect effects.

## 2. Materials and Methods

### 2.1. Study design

**Figure 1.**
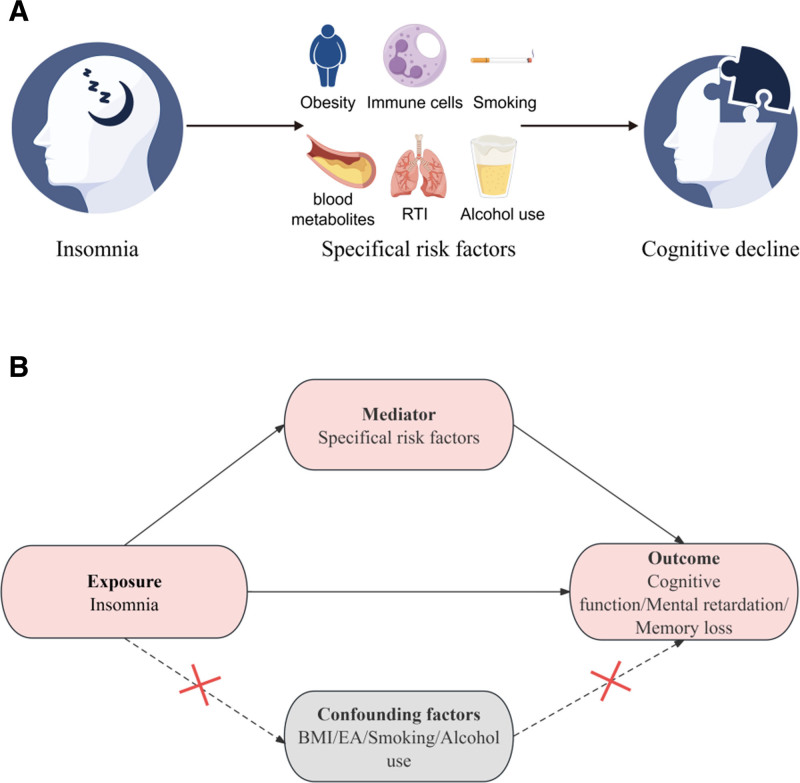
Study design and assumptions for analyses of the relationship between insomnia and cognitive function, and the mediating effects of multiple risk factors. (A) Conceptual pathway: insomnia may contribute to cognitive decline indirectly through several modifiable risk factors: obesity, altered immune cells, smoking, dysregulated blood metabolites, RTIs, and alcohol use. (B) Intermediary analysis chart: insomnia (exposure) influences cognitive outcomes (memory loss, reduced cognitive function, or mental retardation) both directly and through the mediator (specific risk factors shown in A). Dashed arrows indicate potential confounding paths via BMI, EA, smoking, and alcohol use, which are controlled for (red crosses). BMI = body mass index, EA = educational attainment, RTI = respiratory tract infection.

SNPs derived from published insomnia-related GWAS datasets were utilized as insomnia-related IVs, with the causal effect of insomnia on cognitive function being assessed via a 2-sample MR approach.^[13]^ The present 2-sample MR study was developed based on 3 major assumptions: the assumption that IVs are only related to insomnia (relevance), IVs are not associated with confounding factors that have an effect on the relationship between insomnia and cognitive function (independence), and IVs only impact cognitive function through insomnia (exclusion restriction; Fig. 1). This analysis also included BMI, smoking,^[14]^ alcohol intake,^[15]^ and educational attainment (EA)^[16]^ as factors with the potential to confound the relationship between insomnia and cognitive function. An MVMR approach was implemented to adjust for these factors, allowing for the calculation of the causal effect of insomnia on cognitive function.^[17]^ A 2-step MR analysis was then performed to examine the mediating effects of various risk factors on the interplay between insomnia and cognitive decline.

### 2.2. GWAS data

For this study, data were retrieved from publicly available GWAS datasets. However, it is important to note that these datasets did not include specific details regarding disease severity or demographic characteristics (e.g., age, sex, and ethnicity) of the participants.

#### 2.2.1. Insomnia-related GWAS data

A GWAS summary dataset published by Mbatchou et al^[13]^ was used to conduct this study. This dataset included 407,746 samples from the UK Biobank cohort, based in Manchester, United Kingdom, who were 40 to 69 years of age and lived in the United Kingdom from 2006 to 2010. The insomnia status of these patients was assessed by their responses to the following question on a touchscreen-based questionnaire: “Do you have difficulty falling asleep or wake up in the middle of the night?” never, sometimes, often, and possible answer choices were: never, sometimes, often, and refuse to answer. If the participant clicked the button for “help” for this question, they received the following prompt: “If there have been significant changes, please consider the past 4 weeks when answering this question.”

#### 2.2.2. Cognitive function–related GWAS data

A GWAS dataset summarized by the Within Family Consortium that included 22,593 samples served as the source of data related to cognitive function for this study.^[18]^ Principal component analysis of the results of a minimum of 3 cognitive tests assessing different cognitive domains was used to assess cognition, yielding a general cognitive function score. Just 1 score per cognitive test was used, and cognitive functional assessments used to detect dementia in the clinic, such as the Mini-Mental State Examination, were not included. The principal component analysis–derived general cognitive function score was transformed into a standard variable, with higher scores being indicative of better cognitive function.

The GWAS dataset pertaining to memory loss and mental retardation was obtained from the latest results from the tenth round of FinnGen, with respective sample sizes of 393,373 and 303,066 individuals.^[19]^ FinnGen, a large public-private genomics initiative, has analyzed genome-wide and health data from 500,000 Finnish biobank donors to uncover the genetic basis of disease. The International Statistical Classification of Diseases and Related Health Problems (tenth edition) was used to diagnose memory loss and slower cognitive function. All FinnGen study participants, including both patients and controls, participated after providing informed consent. Individuals were excluded from these datasets if they had an uncertain gender, a high genotype missing rate (>5%), high levels of heterozygosity (>±4 standard deviations), cancer, or were of non-Finnish ancestry.^[19]^ Smoking and alcohol consumption–related data were obtained from the UK Biobank to conduct MVMR analyses. BMI- and EA-related data were respectively derived from GWAS datasets summarized by Hoffmann et al^[20]^ and the Social Science Genetic Association Consortium.^[21]^

The GWAS dataset pertaining to RTI incidence, as a potential mediating factor, was obtained from the tenth round of FinnGen^[19]^ and the latest findings published by Hamilton et al.^[22]^ A dataset related to risk factors including BMI, blood metabolites, and immune cells was accessed through an online GWAS database assembled by the Medical Research Council Integrated Epidemiology Unit in the United Kingdom.^[23]^ All of the data used for this study were from individuals of European ancestry. The exposure and outcome datasets were derived from nonoverlapping sample cohorts. Finally, as the data used in this study are publicly available, ethical approval was not required. For further details regarding these datasets, see Table S1, Supplemental Digital Content, https://links.lww.com/MD/P477.

### 2.3. Instrumental variable selection

IVs were those SNPs that were significantly related to insomnia (*P* < 5 × 10^−6^ or relaxed to *P* < 5 × 10^−5^), excluding any SNPs in linkage disequilibrium (r^2^ < 0.001; SNP cluster window > 10,000 kb) to ensure that the selected SNPs were independent of one another.^[24]^ The potential associations between these SNPs and confounding factors (BMI, EA, alcohol intake, and smoking) were assessed with LDlink. Weak IV-related bias was mitigated by calculating F-statistic values for each SNP and eliminating those SNPs with an F-statistic < 10.^[25]^

### 2.4. Statistical analysis

An inverse variance-weighted (IVW) approach was implemented as the main univariate analytical technique in this study, with supplementary approaches including MR-Egger, the weighted median method, the maximum likelihood estimation method, and MR-robust adjusted profile score (MR-RAPS) approaches also being employed for this study. Based on the assumption that all SNPs included as IVs are valid, the IVW method functions by converting Wald ratios for each SNP into the effect of particular risk factors on the outcome.^[26]^ IVW provides the most accurate estimate but ignores invalid IVs and horizontal pleiotropy. MR-Egger regression allowed for the existence of intercept terms and provided a robust causal effect estimate after adjusting for horizontal multiplicity.^[27]^ The weighted median method is capable of generating robust causal effect estimates even if 50% of the included IVs are not valid.^[28]^ The maximum likelihood estimation method takes the uncertainty of SNP-exposure relationships and sample overlap into account in 2-sample MR analyses.^[29]^ The MR-RAPS approach takes specific pleiotropy into account and is capable of overcoming bias associated with weak IVs such that MR analyses can still yield robust inferences.^[30]^ In summary, IVW provided the primary estimate, MR-Egger, and weighted median assessed sensitivity to pleiotropy, and MR-RAPS corrected for weak instruments. Consistency across methods strengthens causal inference.

Following adjustment for BMI, EA, alcohol intake, and smoking, an MVMR analysis was performed to probe the association between insomnia and cognitive decline. Bonferroni correction was used to correct significance thresholds for multiple exposure outcomes, with *P* values below the corrected threshold being considered significant.

The mediation analysis was performed through a 2-step MR approach. Initially, univariate MR analysis was used to estimate the causal effect of insomnia on the mediator variable (β1). Then, the causal effect of this mediator variable on the study outcome was estimated with an MVMR approach (β2). Last, a 2-sample MR approach was used to calculate the total effect between insomnia and the outcome (β0). If β0, β1, and β2 are all significant, this indicates that the exposure and outcome are causally linked, with a partial role for the mediator variable as a regulator of the relationship. The mediation effect is calculated based on β1*β2, while the proportion of the effect mediated by this variable can be computed as β1*β2/β0.^[31]^

The Cochran Q test was used for sensitivity analyses, while the MR-Egger intercept test was used to assess potential horizontal pleiotropy and heterogeneity in the IVW model,^[32]^ with *P* < .05 as the significance threshold. Outliers were detected with the Mendelian Randomization Pleiotropy RESidual Sum and Outlier and MR Radial methods.^[33,34]^ To determine whether causal effects in the IVW analysis were driven by any single SNP, a leave-one-out sensitivity test was performed. The “TwoSampleMR,” “MendelianRandomization,” and “mr.raps” packages in R Studio (v 4.3.1) were used to perform all analyses.

## 3. Results

### 3.1. Univariate MR results

Based on the established criteria for IV selection, SNPs were excluded if they exhibited strong correlations with BMI, EA, smoking, or alcohol consumption or if they exhibited low F-statistics. The remaining SNPs were used to conduct MR analyses with the Mendelian Randomization Pleiotropy RESidual Sum and Outlier and MR Radial methods to eliminate possible outliers.^[33,34]^ All included IVs exhibited F-statistic values >10, indicating that weak instrument bias is unlikely to affect these results.

**Figure 2.**
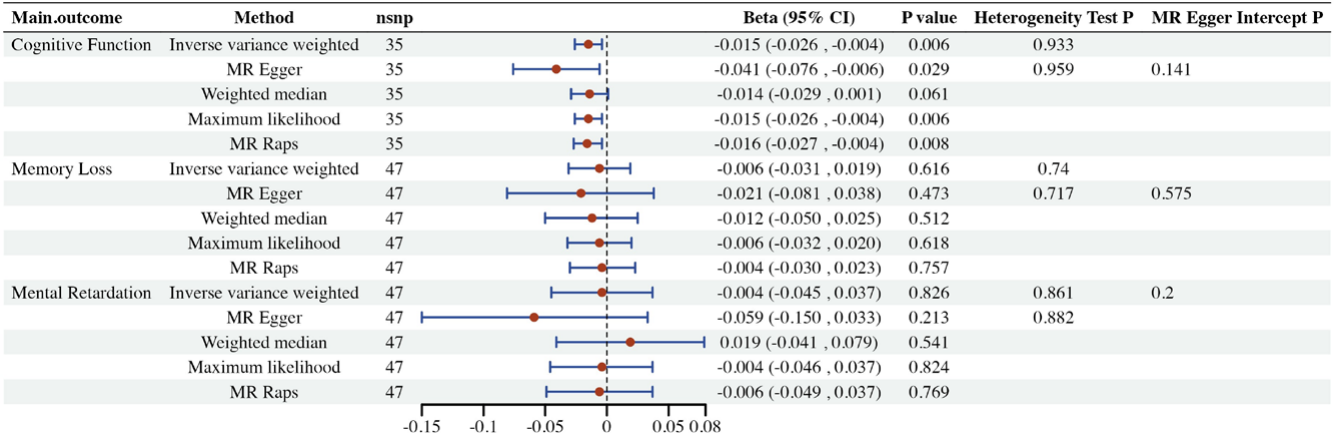
Forest plot corresponding to 2-sample Mendelian randomization (MR) estimates of the impact of insomnia on cognitive function.

Univariate MR analyses revealed a negative causal effect between genetically predicted insomnia and cognitive function (β, −0.015 [95% CI, −0.026 to −0.004]; *P* = .006). Consistent directionality of the total effect values was observed for the IVW, MR-Egger, weighted median, maximum likelihood estimation, and MR-RAPS methods (β < 0). IVW analyses revealed no association between insomnia and either memory loss (β, −0.006 [95% CI, −0.031 to 0.019]; *P* = .616) or mental retardation (β, −0.004 [95% CI, −0.045 to 0.037]; *P* = .826). Following Bonferroni correction (*P* = .05/3 = 0.017), insomnia remained significantly associated with cognitive decline (Fig. 2).

Sensitivity analyses reinforced the robustness of findings. The Cochran Q test (Q = 22.566; *P* = .933) indicated no significant heterogeneity across IVs, supporting the validity of the IVW method’s assumption of homogeneous SNP effects. Moreover, no horizontal pleiotropy was detected through the MR-Egger intercept test (intercept = −0.011; *P* = .141), indicating that the IVs do not impact the study outcomes through pathways other than the exposure. Leave-one-out analyses indicated that no single SNP was a driver of the causal effect.

### 3.2. MVMR

**Figure 3.**
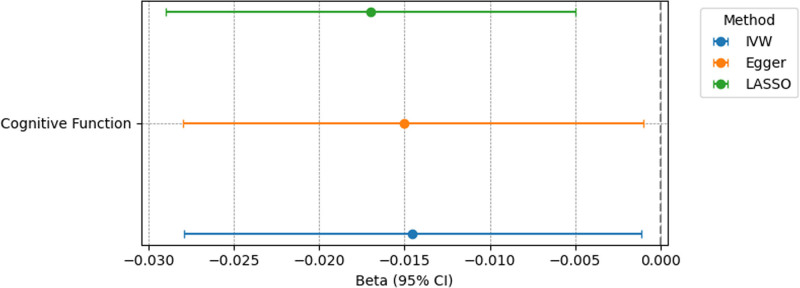
A forest plot representing multivariate Mendelian randomization estimates of the relationship between insomnia and cognitive function following adjustment for confounding factors. IVW = inverse variance-weighted, LASSO = least absolute shrinkage and selection operator.

Following adjustment for BMI, EA, alcohol intake, and smoking, MVMR analyses revealed that insomnia was a potential risk factor associated with cognitive decline (β, −0.015 [95% CI, −0.028 to −0.001]; *P* = .034). Similar results were obtained through the MV-Egger and MV-least absolute shrinkage and selection operator analyses (Fig. 3). The Cochran Q test (*P* < .001) detected heterogeneity when assessing these MVMR results, but the findings were still considered reliable owing to the use of a random-effects IVW model. No horizontal pleiotropy was detected in the MR-Egger intercept analysis (*P* = .733).

### 3.3. Mediation analysis of the association between insomnia and cognitive function

Mediation analyses were next performed with a 2-step approach. Following the initial screening of multiple potentially mediating variables, only RTI was found to exhibit significant effects, whereas other analyzed factors including BMI, immune cell counts, and blood metabolites exhibited no significant mediating effects (*P* > .05). Genetically predicted insomnia was not associated with influenza, acute upper RTIs, or acute lower RTIs, whereas it was significantly associated with pneumonia (β1, 0.072 [95% CI, 0.020–0.125]; *P* = .007) and the common cold (β1, 0.021 [95% CI, 0.003–0.038]; *P* = .023; Table 1). When these 2 forms of RTIs were separately included in the MVMR analysis, the causal effect of insomnia on cognitive decline became weaker (β, −0.009 [95% CI, −0.018 to −9×10^−5^]; *P* = .047), pneumonia had a significant effect on cognitive decline (β2, −0.032 [95% CI, −0.0549 to −0.009]; *P* = .005), and the common cold did not remain significantly associated with cognitive decline (β2, −0.007 [95% CI, −0.017 to 0002]; *P* = .137). Univariate MR analyses indicated that insomnia was significantly negatively correlated with cognitive function, with a total effect size of −0.015. As β0, β1, and β2 are all significant, these data indicate that insomnia is negatively causally related to cognitive function, while pneumonia serves as a partial mediator that explains 15.4% of this relationship.

**Table 1:** Two-step Mendelian randomization estimates for RTI as a mediator of the relationship between insomnia and cognitive function.

	Method	β (95% CI)	SE	*P*_val_
Insomnia-RTI				
Common cold	IVW	0.021 (0.003~0.038)	0.009	.023
Influenza	IVW	0.010 (−0.006 to 0.025)	0.008	.228
Upper respiratory infections	IVW	3×10^−5^ (−0.005 to 0.005)	0.002	.989
Lower respiratory infection	IVW	0.011 (−0.006 to 0.029)	0.009	.215
Pneumonia	IVW	0.072 (0.020 to 0.125)	0.027	.007
RTI-cognitive function				
Common cold	MVMR (adjusted insomnia)	−0.007 (−0.017 to 0002)	0.005	.137
Pneumonia	MVMR (adjusted insomnia)	−0.032 (−0.0549 to −0.009)	0.012	.005
Insomnia-cognitive function				
Insomnia	MVMR (adjusted common cold)	−0.002 (−0.009 to 0.006)	0.004	.666
Insomnia	MVMR (adjusted pneumonia)	−0.009 (−0.018 to −9×10^−5^)	0.004	.047

IVW = inverse variance-weighted, MVMR = multivariable Mendelian randomization, RTI = respiratory tract infection.

## 4. Discussion

The negative correlative relationship between insomnia and cognitive function remains subject to controversy. Adequate sleep quality is essential for good attention, concentration, memory, and executive function. The mechanisms by which insomnia leads to cognitive decline include changes in the neurotransmitter system during sleep, neurological disorders, and hippocampal volume reduction, changes in the function of the hypothalamic-pituitary-adrenal axis, and changes in the function of different brain regions involved in working memory. However, some observational studies have found no significant relationship between insomnia and cognitive decline.^[35]^ Observational study findings are hampered by factors including the potential for reverse causality, challenges associated with patient follow-up, the specific inclusion/exclusion criteria used, and/or the demographic features of the study populations. This study evaluated the causal relationship between insomnia and cognitive function using a 2-sample MR approach and verified the reliability of the results with MVMR. Finally, a 2-step MR method was employed to investigate the potential mediating pathway through which insomnia affects cognitive function. MR analysis addresses these limitations by leveraging genetic instruments to establish a robust causal link. The results revealed that insomnia hinders the improvement of cognitive function, and pneumonia plays a significant mediating role between these 2 factors. Mediation analysis indicated a genetic association between insomnia and an increased risk of pneumonia, which, in turn, contributes to the decline in cognitive function. Notably, pneumonia partially mediates the effect of insomnia on cognitive function, accounting for 15.4% of the association. These findings further supplement the evidence provided by current observational studies.

Insomnia can adversely affect cognitive function through the disruption of a normal sleep-wake balance and circadian rhythm activity. These disruptions lead to altered patterns of gene expression in the cortical regions of the brain associated with cognition and in subcortical regions associated with wakefulness.^[36]^ This can further disrupt neurogenesis and the functional areas of the hippocampus, resulting in symptoms that resemble those associated with the early stages of cognitive impairment.^[37]^ The brain-derived neurotrophic factor (BDNF) plays a key role in neural plasticity, influencing synaptogenesis through the control of synaptic dendritization and morphogenesis, promoting new neuronal circuit formation and improved cognitive performance.^[38]^ Sánchez-García et al^[39]^ determined that insomnia can lower pro-BDNF synthesis and levels of BDNF, thereby contributing to cognitive deterioration irrespective of gender or psychiatric comorbidities.

A 2-step MR approach was employed in this study to investigate whether RTIs mediate the relationship between insomnia and declining cognitive function. In the first step, genetic variants associated with insomnia were used as instruments to assess the causal effect of insomnia on RTI. In the second step, we analyzed whether RTI affects the decline in cognitive function using RTI-related SNPs. Our findings suggest that RTI may act as a mediator in the association between insomnia and cognitive decline, supporting the hypothesis that insomnia-related inflammation may play a pivotal role in cognitive deterioration. Insomnia can trigger inflammation, including the production of higher interleukin-6 and C-reactive protein levels,^[40]^ impaired natural killer cell activity,^[41]^ reduced T-cell cytokine levels,^[42]^ and the inhibition of the i nterleukin-6 response.^[40]^ Insomnia can also delay or weaken vaccine responses.^[43]^ These mechanisms highlight the important role that sleep plays as a regulator of immunity and disease susceptibility. Pneumonia can also provoke the production of cytotoxic amyloid and tau variants by the pulmonary endothelium. In addition to promoting exudative pulmonary edema, thus interfering with the repair of damage, they can also disseminate to the brain via the bloodstream, wherein they can cause direct or indirect damage to long-term hippocampal potentiation and information processing, reducing brain neuron dendritic spine density, and thereby leading to cognitive decline.^[44,45]^ For individuals with chronic insomnia, preventive measures should be prioritized, including lifestyle modifications and, when appropriate, vaccination against pneumococcus and influenza to reduce the incidence of RTI and downstream neuroinflammatory cascades.^[46]^ Clinically, an integrated approach involving sleep specialists, pulmonologists, and neurologists may help address overlapping pathways such as neuroinflammation and immune dysregulation in patients with comorbid insomnia and cognitive impairment. Finally, routine cognitive screening should be implemented for individuals with chronic insomnia to enable the early detection of cognitive decline.

While several risk factors including BMI and immune cells may be biologically related to insomnia and cognitive functionality, they were not identified as significant mediators in the present study based on the available data. This reflects subtle methodological or biological differences. The relationship between BMI and cognitive decline may be nonlinear or contingent upon comorbid conditions (such as diabetes), and the assumption of linearity in MR analyses may overlook these complexities.^[47]^ Similarly, while the quantity of immune cells is associated with neuroinflammation, their pleiotropic effects, where subgroups (e.g., proinflammatory cells and regulatory T cells) may counterbalance one another, can result in a net effect of zero.^[48]^ Furthermore, blood metabolites (e.g., glucose and blood lipids) may require prolonged exposure or synergistic interactions (such as with insulin resistance) to mediate cognitive decline.^[49]^ The null findings regarding these factors in the present study suggest that future research will require larger GWAS datasets incorporating detailed phenotypic data, such as immune cell subtypes and longitudinal metabolomic profiles, to uncover their potential modulatory roles.

All of the data used to conduct this study were from individuals of European ancestry, thereby minimizing the potential for population stratification bias. Despite these efforts to maximize result validity, this study is subject to some limitations. For one, looser thresholds (*P* < 5 × 10^−6^ to *P* < 5 × 10^−5^) were used when extracting IVs to improve overall statistical power, but this has the potential to introduce weak instrument bias, violating the assumptions necessary to conduct MR analyses. All of these IVs exhibited F-statistic values >10, and the MR-RAPS method was employed to correct for weak instrument bias to maximize result reliability. Sensitivity analyses, including MR-Egger and leave-one-out tests, demonstrated consistent effect estimates across methods, further supporting the reliability of our results. Similar strategies have been successfully employed in prior MR studies of sleep-related traits.^[24]^ However, the findings should still be interpreted with caution. Second, all of the samples in this study were derived from a single ethnic population such that their generalizability to other populations remains to be assessed and may miss the discovery of population-specific variants. Future studies should actively include participants from various ancestral backgrounds to enhance the representativeness of findings. If necessary, use analytical methods that take into account population stratification and genetic diversity to ensure that the results are reliable and applicable to multiple groups. Finally, no details regarding disease severity or demographics were available for the utilized GWAS datasets, precluding any subgroup analyses. While our findings suggest a significant relationship between insomnia and cognitive decline, the specific mechanisms underlying this association remain to be fully elucidated. Therefore, future research should more thoroughly interrogate the mechanistic link between insomnia and cognitive decline to enhance our understanding and guide effective interventions.

## 5. Conclusion

In conclusion, an MR approach was, herein, used to explore the causal relationship between insomnia and cognitive decline, identifying pneumonia as an RTI that was a partial mediator of this causal relationship. Therefore, prioritizing cognitive behavioral therapy for insomnia, vaccination programs, and interdisciplinary care can directly improve cognitive outcomes in individuals with insomnia while also indirectly mitigating pneumonia-related neurotoxicity. Future studies should build on these results by exploring other factors that may mediate the complex interplay between insomnia and cognitive function, thereby providing a basis for future interventional strategies.

## Acknowledgments

The authors would like to express their sincere gratitude to the Within Family GWAS Consortium and the UK Biobank Consortium for generously providing the original data used in the Mendelian randomization analyses. The authors also want to acknowledge the participants and investigators of the FinnGen study. FinnGen is a research project in genomics and personalized medicine. It is large public-private partnership that has collected and analysed genome and health data from 500,000 Finnish biobank donors to understand the genetic basis of diseases.

## Author contributions

**Conceptualization:** Zilin Wang, Zihan Qu, Hongshi Zhang.

**Data curation:** Zilin Wang.

**Formal analysis:** Zilin Wang, Jiawei Yin, Hongshi Zhang.

**Writing – original draft:** Zilin Wang.

**Investigation:** Zihan Qu.

**Methodology:** Zihan Qu.

**Supervision:** Zihan Qu, Yuqing Song, Jiawei Yin.

**Project administration:** Jindan Zhang.

**Software:** Jindan Zhang, Jiawei Yin.

**Validation:** Jindan Zhang, Yuqing Song, Jiawei Yin, Hongshi Zhang.

**Writing – review & editing:** Yuqing Song, Hongshi Zhang.

**Funding acquisition:** Hongshi Zhang.

## Supplementary Material


