# Supplementary material for: The causal association between insomnia and cognitive decline: A 2-sample, 2-step multivariable Mendelian randomization study
Source: Medicine (Baltimore). 2025 Jul 18;104(29):e43417. doi: 10.1097/MD.0000000000043417 (PMC12282771; doi:10.1097/MD.0000000000043417)
Supplement: Supplementary file 1 [file medi-104-e43417-s001.pdf]

Table S1.Information of the datasets used in this study in details

Data Source	Phenotype	Year	Simple size (case/control)	Population	Adjustment
UKB	Insomnia	2021	486,627	European	Sex, genotyping arrays, and the top 10 principal components
	Smoking	2018	425,516	European	
	Alcohol use	2018	463,010	European	
FinnGen	Memory loss	2023	393,373	European	Unclear sex, High genotypic deletion rates (>5%), Excessive heterozygosity (±4 SDs), Other cancers, and non-Finnish ancestry
	Mental retardation	2023	303,066	European	
	Common cold	2023	339,462	European	
	Influenza	2023	349,544	European	
	Upper respiratory infections	2023	412,181	European	
	Lower respiratory infection	2023	394,416	European	
WFC	Cognitive function	2022	22,593	European	Sex,Genetic outliers,Low genotyping rate and non-European ancestry.
SSGAC	Education attainment	2016	293,723	European	Age,Sex,Genetic outliers,poor genotyping rates and non-European ancestry.
Hoffmann et al.	BMI	2018	315,347	European	Age,Sex,Outlier BMI measurements,childbirth,bariatric surgeryand non-European ancestry.
Hamilton et al.	Pneumonia	2021	431,365	European	Sex, genotyping arrays, and the top 10 principal components
Sun et al.	Protein levels	2018	3,301	European	Sex mismatches, Low call rates, Duplicate samples, Extreme heterozygosity and non-European descent.
Elliott et al.	Brain region volumes	2018	8,428	European	Minor allele frequency (MAF) below 0.1% and with an imputation information score below 0.3,non-European descent and Hardy–Weinberg equilibrium P value <10−7

Table S1.Information of the datasets used in this study in details

Data Source	Phenotype	Year	Simple size (case/control)	Population	Adjustment
Shin et al.	Blood metabolites	2014	7,822	European	Sex,Age,SNPs with low imputation quality (info<0.4), low minor allele frequency (<0.01), significant heterogeneity of effects between the two cohorts or present in only one cohort.
Roederer et al	Immune cells	2015	497	European	Sample call rate <98%, Heterozygosity across all SNPs ≥2 standard deviation (SD) from the sample mean, non-European ancestry, pairwise identity by descent

Table S2. The characteristics of the selected SNPs on insomnia.

SNP	Chr	Pos	Effect allele	Other allele	EAF	P-value	Beta	SE	R^2	F
rs61801303	1	168453624	G	A	0.1541	4.25E-05	-0.443643	0.112392	0.051312	355.65296
rs1751340	1	201578110	T	G	0.0219	3.14E-05	0.806482	0.170599	0.0278642	188.47368
rs72701210	1	107954172	T	C	0.0119	4.00E-05	1.00881	0.210519	0.023933	161.23075
rs10929027	2	235229471	G	A	0.5636	3.47E-05	-0.294618	0.0699634	0.0426977	293.28245
rs75352062	2	6299464	C	T	0.0129	1.20E-05	1.20832	0.232978	0.037183	253.9405
rs78749835	2	66181023	A	G	0.0765	1.74E-05	0.470354	0.10218	0.0312592	212.17848
rs140866347	2	186000808	T	A	0.0467	3.86E-05	0.677197	0.14867	0.0408326	279.92585
rs16833234	3	173810957	T	C	0.2078	3.16E-05	-0.461653	0.115409	0.0701684	496.21321
rs55993471	3	103914948	C	A	0.5199	1.22E-05	0.322479	0.0729998	0.051914	360.05394
rs13072401	3	33972533	G	A	0.4781	7.46E-06	0.324199	0.0713157	0.0524517	363.98956
rs9848693	3	194588619	A	C	0.9344	9.16E-06	-0.634149	0.129679	0.0493003	340.98622
rs116592488	3	56347420	T	C	0.0417	4.77E-06	0.772152	0.14976	0.0476511	329.00915
rs72656212	4	86552106	G	A	0.0159	5.32E-07	1.11831	0.188936	0.0391373	267.83069
rs6830345	4	187005376	C	T	0.7565	4.99E-05	0.366502	0.0921238	0.0494869	342.34455
rs6825948	4	111168654	T	C	0.3678	4.44E-05	0.298867	0.0713162	0.0415386	284.9761
rs77682150	5	36665842	G	C	0.0497	2.68E-05	0.643937	0.138733	0.0391682	268.051
rs10058494	5	23130841	G	A	0.1769	2.18E-05	-0.469436	0.114974	0.0641746	450.91979
rs10039772	5	141758597	G	T	0.5487	1.50E-05	0.314926	0.0721144	0.0491188	339.6659
rs116735820	6	32027446	T	A	0.0119	2.88E-05	1.19876	0.239164	0.0337942	229.98718
rs9350315	6	21055717	C	G	0.332	2.90E-05	-0.333851	0.0803623	0.0494367	341.97928
rs1611255	6	29751005	A	C	0.1382	1.39E-05	-0.536161	0.126179	0.0684755	483.36097
rs72946490	6	109050170	T	C	0.0577	1.89E-05	0.634209	0.134952	0.0437381	300.75569
rs2922249	6	127954614	C	A	0.5328	2.51E-06	-0.337753	0.0703888	0.0567931	395.93092
rs9347311	6	159781828	T	C	0.3608	3.72E-05	-0.322851	0.0785836	0.048077	332.09831
rs9361123	6	77458473	A	G	0.2694	2.70E-05	-0.384901	0.0933239	0.0583184	407.22286
rs72952803	6	120215516	T	C	0.0139	8.10E-06	0.991493	0.1905	0.0269491	182.11278
rs1404924	7	110309145	A	G	0.4801	4.13E-05	-0.307919	0.074491	0.047332	326.696
rs55982055	7	66919087	G	C	0.501	2.86E-05	0.307551	0.0728974	0.0472936	326.41822
rs12702120	7	45215953	C	T	0.2734	1.96E-05	-0.363703	0.0863556	0.0525554	364.74956
rs28399916	7	73263055	C	T	0.4095	6.98E-06	0.372315	0.0811894	0.0670386	472.48944
rs6962111	7	134795986	C	T	0.1153	3.33E-05	-0.485066	0.122159	0.0480017	331.55208
rs149697421	7	155279172	C	T	0.0378	3.46E-05	0.58127	0.128287	0.0245778	165.68426
rs2458	8	134468066	A	G	0.2018	2.45E-05	-0.397463	0.0964085	0.0508928	352.59158
rs59397035	8	119622950	C	A	0.2435	4.60E-06	-0.410858	0.0912336	0.0621901	436.05121
rs7822651	8	73806225	T	C	0.5119	8.08E-06	0.325444	0.0721027	0.0529269	367.47169
rs568083869	8	1823301	T	C	0.0229	1.35E-05	0.784212	0.160146	0.0275215	186.0897
rs12683347	9	19898468	T	C	0.2614	2.58E-05	0.326657	0.0749657	0.041203	282.57456
rs7848365	9	116120186	C	A	0.327	8.64E-07	0.361444	0.0714798	0.0575009	401.16666
rs114709635	9	139565488	C	G	0.4125	3.88E-05	0.32485	0.0777431	0.0511479	354.45405
rs148106195	10	80244515	T	C	0.0278	3.63E-05	0.660127	0.144445	0.0235551	158.62389
rs7914098	10	3498220	C	G	0.0109	1.53E-05	1.41396	0.266792	0.0431093	296.23715
rs77074742	11	19020397	G	T	0.0308	1.05E-05	0.781622	0.157276	0.0364744	248.91743
rs4363570	11	7504815	C	T	0.5199	2.63E-05	0.302434	0.0710607	0.0456607	314.60879
rs188056810	11	130048874	T	C	0.0119	4.00E-07	1.55885	0.24728	0.0571461	398.54101
rs61578011	12	43047117	T	G	0.0298	8.12E-06	1.34217	0.252855	0.1041652	764.58462
rs1515773	12	20871072	T	C	0.2147	1.88E-05	0.349313	0.0782713	0.041146	282.16656
rs78384438	14	98160042	A	G	0.0268	1.36E-05	0.770131	0.157752	0.0309383	209.93052
rs75951922	14	24087979	C	T	0.0537	2.24E-05	-0.993429	0.268516	0.1003014	733.06188
rs117175090	14	62796559	C	A	0.0169	3.77E-05	0.947919	0.198858	0.0298577	202.37289
rs113446181	15	29025532	A	G	0.0487	6.72E-06	-1.3603	0.349767	0.1714533	1360.6918
rs1134445	15	31515046	A	G	0.0328	3.67E-05	0.584925	0.12949	0.021708	145.90928
rs118058981	15	69356779	G	A	0.0268	2.69E-05	0.728173	0.15471	0.027659	187.04596
rs186967203	16	63950333	A	C	0.0169	2.84E-05	1.18788	0.238391	0.0468878	323.47923
rs2239335	16	22897795	A	G	0.0328	2.89E-05	0.683404	0.14694	0.029633	200.80293
rs4429282	16	7987471	T	C	0.837	6.76E-06	-0.421675	0.0885226	0.0485175	335.2964
rs150442603	16	9815773	C	T	0.0189	4.99E-05	0.931503	0.199277	0.0321791	218.62987
rs16955182	17	52913104	T	A	0.0586	3.21E-05	0.523912	0.116331	0.0302844	205.35487
rs9807431	18	75109503	G	A	0.0696	1.62E-05	0.496828	0.107084	0.0319684	217.15131
rs142593043	19	5613450	T	C	0.0109	1.66E-05	1.0597	0.20952	0.0242138	163.16943
rs117232445	19	6215063	T	C	0.0368	3.97E-05	0.665415	0.146564	0.0313891	213.08901
rs6032802	20	10115599	A	C	0.6103	4.88E-05	0.30492	0.0750172	0.0442258	304.26444
rs211961	21	18951018	A	G	0.1362	7.72E-06	-0.525421	0.122417	0.0649584	456.8095

SNP, single-nucleotide polymorphism; Chr, chromosome; pos, position; Beta, estimate of the effect of the association; SE. standard error; R^2, percentage of the variation of insomnia explained by the SNP; F, F-statistic.
